# Biotic and abiotic drivers affect parasite richness, prevalence and abundance in *Mytilus galloprovincialis* along the Northern Adriatic Sea

**DOI:** 10.1017/S0031182021001438

**Published:** 2022-01

**Authors:** C. Bommarito, M. Wahl, D.W. Thieltges, C. Pansch, M. Zucchetta, F. Pranovi

**Affiliations:** 1 Department of Marine Ecology, GEOMAR Helmholtz Centre for Ocean Research Kiel, Hohenbergstr. 2, 24105, Kiel, Germany; 2 Department of Coastal Systems, NIOZ Royal Netherlands Institute for Sea Research, P.O. Box 59, 1790, AB Den Burg Texel, The Netherlands; 3 Environmental and Marine Biology, Åbo Akademi University, Artillerigatan 6, 20520 Åbo, Finland; 4 Institute of Polar Sciences, ISP-CNR, Via Torino 155, 30172 Venice-Mestre, Italy; 5 Department of Environmental Sciences, Informatics and Statistics, University Ca’ Foscari of Venice, Via Torino 155, 30172, Venice, Italy

**Keywords:** Abundance, Adriatic, eutrophication, *Mytilus galloprovincialis*, parasite, prevalence, richness, TRIX

## Abstract

Although it is generally known that a combination of abiotic and biotic drivers shapes the distribution and abundance of parasites, our understanding of the interplay of these factors remains to be assessed for most marine host species. The present field survey investigated spatial patterns of richness, prevalence and abundance of parasites in *Mytilus galloprovincialis* along the coast of the northern Adriatic Sea. Herein, the relationships between biotic (host size, density and local parasite richness of mussel population) and abiotic (eutrophication and salinity) drivers and parasite richness of mussel individuals, prevalence and abundance were analysed. Local parasite richness was the most relevant factor driving parasite species richness in mussel individuals. Prevalence was mainly driven by eutrophication levels in three out of four parasite species analysed. Similarly, abundance was driven mainly by eutrophication in two parasite species. Mussel size, density and salinity had only minor contributions to the best fitting models. This study highlights that the influence of abiotic and biotic drivers on parasite infections in mussels can be differentially conveyed, depending on the infection measure applied, i.e. parasite richness, prevalence or abundance. Furthermore, it stresses the importance of eutrophication as a major factor influencing parasite prevalence and abundance in mussels in the Adriatic Sea.

## Graphical abstract

**Figure:**
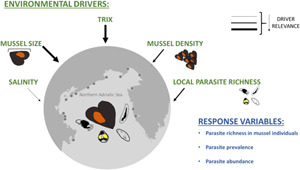

## Introduction

Parasites are increasingly recognized as important ecological players in marine ecosystems (Marcogliese, 2004; Poulin *et al*., 2014; Sures *et al*., 2017), affecting individual hosts through castration and by limiting their growth (de Montaudouin *et al*., 2012). Parasites also influence host reproductive output and can provoke mass mortality events (Fredensborg *et al*., 2005; Thieltges, 2006*a*). The dominant parasites in coastal marine systems are digenean trematodes, which exhibit complex life cycles usually involving snails as first intermediate host, macroinvertebrates, such as bivalves and crustaceans, or vertebrates as second intermediate hosts and fish, birds or mammals as final hosts (Werding, 1969; Galaktionov *et al*., 2006). Bivalves such as mussels of the genus *Mytilus* are common intermediate hosts for trematodes and other symbionts, such as hydroids or turbellarian (Lauckner, 1983). The latter groups may shift from commensalistic to parasitic behaviour when sudden environmental changes occur (Villalba *et al*., 1997; Rayyan *et al*., 2004; Mladineo *et al*., 2012). In the case of turbellarians, deleterious effects can occur in the form of lower condition of infected hosts (Galinou-Mitsoudi *et al*., 2002; Rayyan *et al*., 2004), thus they can be considered parasitic. Despite the diverse influences of parasites on their hosts, multiscale drivers regulating parasite richness, prevalence and abundance in bivalve intermediate hosts remain to be fully understood.

In general, the richness and infection levels of parasites in hosts result from a combination of biotic and abiotic drivers. Among biotic drivers, host size (and age) is generally known to be positively associated with parasite species richness and infection levels in host individuals (Poulin, 2004; Thieltges and Reise, 2006; Thieltges, 2007; Galaktionov *et al*., 2015). As described by Nikolaev *et al*. (2006) and Mouritsen *et al*. (2003), who investigated the pattern of trematode infection in bivalves, larger host individuals provide more space and niches for parasite infections and as they display higher filtration activities, their exposure to infective stages is higher than in smaller individuals. Furthermore, in bivalves, size is usually positively correlated with age, hence larger and older bivalves have a higher chance to accumulate parasites during their lifetime (Nikolaev *et al*., 2006). Another important biotic factor for infection levels among bivalves is the immunity status (Zannella *et al*., 2017). Previous studies showed bivalves haemocytes to display chemoattraction towards products released by infectious agents such as trematodes (Cheng *et al*., 1974; Allam and Raftos, 2015). Changes in the morphology and functions of haemocytes appeared to be correlated with bivalves’ resistance against pathogens (Allam and Raftos, 2015). Abiotic factors such as temperature, for instance, might lower the immunity of the hosts and, consequently, their resistance to diseases (Cherkasov *et al*., 2007).

A well-known biotic driver of parasite infection is the density of host populations. Given a pool of parasites in a location, their richness, prevalence and abundance are directly affected by host population density through higher transmission rates (May and Anderson, 1979; Dobson, 1990; Arneberg, 2002). Parasite richness in individual hosts (infra-community richness) is usually positively related to the total richness of parasites species found in the host species at a specific location (local richness or component community richness; Bush *et al*., 1997; Poulin, 1998). The latter sets a ceiling for the maximum number of parasite species found in an individual host, but this upper limit is rarely realized. However, local species richness represents the pool of parasite species from which hosts can become infected and the more diverse the local pool the more likely it will be to find a more diverse parasite community in individual hosts (Poulin, 1997).

Besides these biotic drivers, several environmental factors may affect parasite richness, prevalence and abundance. Among those, salinity and eutrophication have been identified as very relevant in aquatic ecosystems (Mouritsen, 2002; Johnson *et al*., 2007; Studer and Poulin, 2012). Positive or negative correlations among parasite richness and salinity highly depend on the host–parasite system investigated (Schmidt *et al*., 2003; Blanar *et al*., 2011). As reported by Schmidt *et al*. (2003), most of the parasite taxa found in estuaries are of marine origin. Therefore, their distribution may be limited by low salinity regimes. Yet, the mechanisms underlying the correlation among parasite prevalence and abundance and salinity may slightly differ from those of richness. Previous experimental studies highlighted the prominent role of salinity in the transmission of trematodes’ free-living stages (Koprivnikar and Poulin, 2009; Koprivnikar *et al*., 2010; Lei and Poulin, 2011; Studer and Poulin, 2012; Bommarito *et al*., 2020*a*), showing reduced transmission at lower salinities caused by osmotic stress of both the parasite and its host. Lower transmission may, in turn, results in lower prevalence and abundance of parasites in the target host (Bommarito *et al*., 2020*b*).

Beside salinity, eutrophication also affects parasites richness in aquatic ecosystems. In eutrophic waters, few host species tend to dominate, limiting host diversity and, therefore parasites richness (Budria, 2017). With a reduction in host diversity, generalist parasite species or species with direct development appear favoured (Palm and Dobberstein 1999; Kesting and Zander, 2000; Budria, 2017). Eutrophication can also have effects on parasite prevalence and abundance by increasing abundance of hosts (Zander and Reimer, 2002) and by promoting vegetation growth, and thus physical barriers, which can exert dilution effects on parasite transmission (Thieltges *et al.*, 2008; Prinz *et al*., 2009). In addition, higher nutrients may be assimilated by the parasite (Budria, 2017). A positive effect of eutrophication on trematodes was experimentally confirmed by Johnson *et al*. (2007), who observed parasite transmission to be promoted by two different eutrophication-related mechanisms: (1) an increase in first intermediate host size and robustness directly led to an increase in the production of trematode cercariae and (2) an increase in growth, reproduction and survival of herbivorous hosts, also increases the availability of intermediate hosts and, in turn, lead to an increase in density of infected hosts.

The present study focuses on parasites of *Mytilus galloprovincialis* in the North Adriatic Sea. This bivalve is ubiquitous in the Mediterranean Sea and important from both an ecological and a commercial perspective. Parasites such as trematodes are known to impact growth and the quality of the aquaculture product (Buck *et al*., 2005) and financial losses from infections, such as from growth retardation, mortality of the stock and veterinary costs, can be substantial (Paladini *et al*., 2017). Mussels of the genus *Mytilus* serve as first or second intermediate host for a vast number of digenean trematodes worldwide such as Gymnophallidae or Renicolidae (Lauckner, 1983; Galaktionov *et al*., 1996) and for hydrozoan and turbellarian species (Piraino *et al*., 1994; Boero and Bouillon, 2005; Mladineo *et al*., 2012). To date, very few studies focused parasite-host relationships in the Adriatic Sea, the majority investigating on parasites in fishes (Mladineo, 2005; Smrzlić *et al*., 2012; Bušelić *et al*., 2018; Mladineo *et al*., 2020) and only very few parasites in bivalve molluscs (Piraino *et al*., 1994; Mladineo *et al*., 2012). Moreover, to our knowledge, none of these studies focused on the influence of combined environmental drivers on the interaction between bivalve hosts and parasites in this region. The Adriatic Sea and, in general, the whole Mediterranean Sea, is considered hotspots for global change, since the effects of the latter are combined with local climate variations (Grbec *et al*., 2015). The results of studies on combined stressors in this region might represent valid indicators of global change effects in future, which can be extended to other regions similarly affected by those stressors. By conducting a large field survey of parasite infections in mussels from the eastern to the western coast of the northern Adriatic Sea, the present study aimed (1) to investigate spatial patterns of richness, prevalence and abundance of the parasite community infecting *M. galloprovincialis*, and (2) to explore the biotic (mussel size, mussel population density, local parasite richness) and abiotic (salinity, eutrophication) correlates of parasite species richness, prevalence and abundance found in individual mussels.

## Methodology

### Study area

The Adriatic basin can be divided into three regions, southern, central and northern. The northern region is very shallow, with an average depth of 35 m (Artegiani *et al*., 1997). The western side receives many river runoffs (e.g. Po, Adige, Brenta and Piave rivers), which cause a local decrease in salinity, especially during spring and autumn (Russo *et al*., 2012). In contrast, the eastern side is characterized by the warm and highly saline Eastern Adriatic current (Giani *et al*., 2012). A wind-driven circulation of water masses (Kuzmić and Orlić, 2006) together with the Po River discharge, results in a west-east gradient of nutrients (Solidoro *et al*., 2009). Hence, the western area is characterized by eutrophic waters, suitable for mussel farming (Rampazzo *et al*., 2013), while the east is characterized by oligotrophic waters (Hopkins *et al*., 1999).

### Spatial sampling

**Fig. 1.**
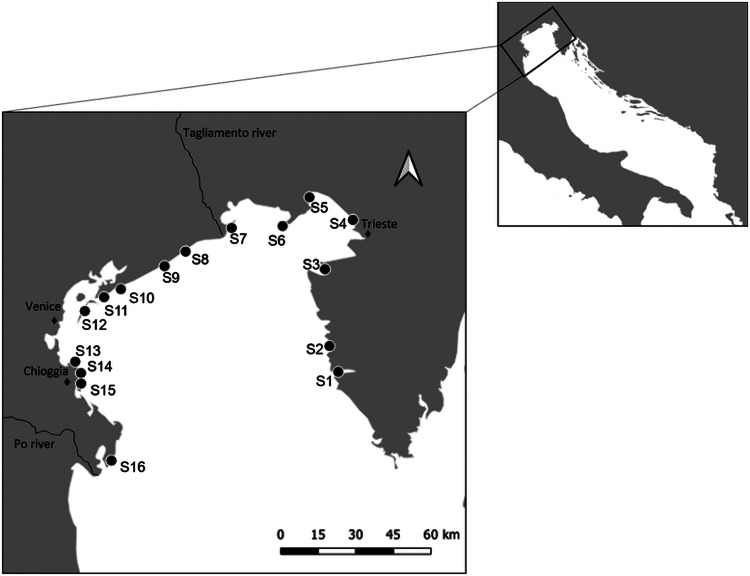
Map of the sampling stations along the North Adriatic Sea. In the western area salinity decreases (surrounding area of the Po Estuary). A wind-driven circulation of water masses together with the Po River discharge results in a west-east gradient of nutrients: The western area of the northern region is characterized by eutrophic waters while the eastern area is characterized by oligotrophic waters.

Sampling was carried out in the northern region of the Adriatic Sea, between November and December 2018, at 16 stations spread from the Po Delta in the west to Istria in the east (Fig. 1; Table 1). The stations were selected based on geographical distribution along the coast (excluding the lagoons), and on logistic feasibility. At each station, a total of 20 individuals of *M. galloprovincialis* were randomly collected at a depth range of 10–30 cm. Mussel density in each station was recorded by counting all individuals in three independent 50 × 50 cm plots, distanced by 25 m, along a 50 m transect of rocky substrate. When the density was higher than 200 individuals per plot, numbers were rounded to the nearest decimal.

**Table 1.** Station name, average salinity, average temperature, average trophic index TRIX (indicator of trophic status of coastal waters), mussel mean density (±s.d.) and average length (±SE) of mussels collected in each station along the North Adriatic Sea

Station	Salinity (psu)	Temperature (°C)		TRIX	Mean mussel density (no. per 50 × 50 cm plot) ± s.d.	Mean mussel size (mm) ± s.e.
		November/December	June/July/August			
S1	38.4 ± 0.4	14.7 ± 2.3	25.4 ± 1.6	4.4	30 ± 0	40.8 ± 2.2
S2	38.5 ± 0.4	14.9 ± 2.2	25.4 ± 1.6	2.7	14.6 ± 10	44.5 ± 0.8
S3	38.1 ± 0.6	14.1 ± 2.5	25.7 ± 1.8	3.8	415 ± 512	30.2 ± 0.6
S4	38.2 ± 0.5	13.5 ± 2.6	25.8 ± 1.9	3.42	156 ± 168	42.9 ± 0.9
S5	38.1 ± 0.5	13.3 ± 2.6	26.0 ± 1.9	4.47	33.3 ± 16	36.9 ± 0.8
S6	38.0 ± 0.6	13.7 ± 2.5	26.2 ± 1.8	4.07	26.6 ± 12	36.6 ± 0.9
S7	37.8 ± 0.7	13.4 ± 2.6	26.5 ± 1.9	4.35	300 ± 50	37.8 ± 0.8
S8	37.7 ± 0.9	13.3 ± 2.7	26.4 ± 1.7	4.2	466.6 ± 57	35.4 ± 0.9
S9	37.5 ± 1.2	13.5 ± 2.5	26.3 ± 1.8	4.7	600 ± 100	36.8 ± 0.8
S10	37.1 ± 1.3	13.5 ± 2.4	26.4 ± 1.9	4.8	1000 ± 0	23.4 ± 0.3
S11	36.8 ± 1.4	13.6 ± 2.4	26.4 ± 1.9	4.5	500 ± 0	30.9 ± 0.9
S12	36.7 ± 1.3	13.4 ± 2.5	26.5 ± 1.9	4.7	523.3 ± 25	36.1 ± 1.2
S13	36.5 ± 1.6	13.7 ± 2.5	26.8 ± 1.7	4.3	236.6 ± 32	36.4 ± 0.9
S14	33.3 ± 2.2	14.4 ± 2.5	26.7 ± 1.7	5.4	800 ± 100	34.1 ± 0.8
S15	33.3 ± 2.2	14.4 ± 2.5	26.2 ± 1.7	5.5	226.6 ± 40	29.4 ± 0.5
S16	27.05 ± 2.4	13.3 ± 2.5	26.2 ± 1.9	6.4	5 ± 0	30.0 ± 1.9

Salinity is reported based on the annual averages of 2018 (±s.d.); temperature on the average of November and December in 2018 (±s.d.), i.e. the period in which the sampling was conducted, and on the average for the summer months (June, July, August), the most relevant for parasite transmission. (Copernicus dataset, http://marine.copernicus.eu).

### Parasite and host analysis

After collection, samples were transferred to the laboratory where the shell length of each individual was recorded. Then, mussels were dissected separating the organs and the tissue of each individual was squeezed between two thick glass slides. Internal mussel organs were macroscopically and microscopically analysed. Parasite identification followed previous parasite descriptions (Bartoli, 1965; Davey and Gee, 1988; Mladineo *et al*., 2012; Özer and Güneydağ, 2014) and based on morphological identification for the metacercariae and morphological and behavioural features for *Eugymnanthea inquilina*, *Urastoma cyprinae* and *Mytilicola* sp. All individual parasites found in a mussel were counted.

### Abiotic parameters

Salinity and temperature data for all the stations were retrieved from the Adriatic Sea physics reanalysis product provided by Copernicus Marine Environment Monitoring Service (http://marine.copernicus.eu). The data were extracted for a water depth of 1.5 m. Annual average salinity and the average temperature for the months November and December 2018 were calculated for each station. Since temperature shows a strong seasonal cycle (Russo *et al*., 2012) we chose to not consider the yearly average but to calculate the average temperature for the 2 months when the sampling took place (November, December). However, since trematode cercariae production and infection are highly occurring in summer, we also calculated the average summer temperature (June, July, August) and verified its variation among sampling station (Table 1).

To estimate eutrophication, the trophic index (TRIX) (Vollenweider *et al*., 1998) was used. This index is composed of four variables connected to primary production: chlorophyll-a, oxygen, dissolved inorganic nitrogen and total phosphorus (for TRIX calculation see Vollenweider *et al*., 1998). The range goes from <4 (low eutrophication) to >6 (high eutrophication). Annual average of 2018 TRIX for each station was provided by the *Agenzia Regionale per la Protezione dell'Ambiente del Veneto (ARPAV)*, by the *Agenzia Regionale per la Protezione dell'Ambiente del Friuli-Venezia Giulia (ARPA FVG)*, by the *Rudjer Boskovic Institute, Slovenia* and by the *Institute of Oceanography and Fisheries, Croatia*. When the stations of the agencies were not exactly matching with the sampling stations, the data of the agency' station in the nearest proximity of the sampling station were considered.

### Data analysis

For an overview of parasite richness, prevalence and abundance at the different sampling stations we calculated: (1) local parasite richness as the total number of parasite species found at a sampling location, and (2) mean parasite richness per individual mussel as the number of parasite species per mussel individual, including infected and not infected individuals (infra-community richness). Where species identification was not possible, individuals were grouped into larger taxa (ciliates, nematodes, copepods). We further calculated (3) prevalence as the percentage of mussels infected with one or more individuals of a particular parasite species, and (4) mean abundance as the number of parasites of a particular species per mussel individual, including infected and not infected individuals.

Our further analyses to investigate the effect of biotic (mussel size, mussel population density, local parasite richness) and abiotic (salinity, TRIX) drivers of infections focussed on individual mussels. We considered prevalence as presence/absence (calculated as 1 or 0) of a parasite species per individual mussel (proportion of infected individuals, between 0 and 1), and abundance as the number of parasites of a certain species in the individual mussel, considering infected and not infected hosts. Individual richness was considered to be the number of parasite species in an individual mussel and thus different from local parasite richness (used as predictor, see above).

To investigate the correlation between individual parasite species richness and predictors, a zero truncated generalized linear mixed model (GLMM) with Poisson distribution was performed, including sampling station as a random factor. For the four main parasite species, the correlation between prevalence and the different predictors was investigated performing GLMMs with a binomial distribution (package glmmTMB; Brooks *et al*., 2017). For the most abundant parasite species, *Parvatrema duboisi* (Gymnophallidae) and *E. inquilina* (Eirenidae), the relationship between the same predictors and abundance was studied performing GLMMs with a negative binomial distribution (package glmmTMB; Brooks *et al*., 2017). The choice of the distribution family was based on a preliminary analysis exploring the residuals on a saturated model using the function ‘simulateResiduals’ (package DHARMa; Hartig, 2020). Abundances of *Mytilicola* sp. and *U. cyprinae* were not included in the analysis due to too frequent zeros in the dataset. The predictors considered for prevalence and abundance were the same as for individual parasite richness except of local parasite richness. We decided to use a polynomial quadratic function for the TRIX data, since within the range of variability considered of the data a linear relationship was not expected. Temperature was not included as a factor due to the low variability observed along the region, both in November/December (13.3–14.9°C) and during summer (June–August; 25.4–26.8°C; Table 1).

The model selection followed the same procedure for richness, prevalence and abundance but for prevalence and abundance all models were performed separately for each parasite species. The first model was fitted including all predictors. Collinearity among predictors was checked through the ‘check_collinearity’ function (package ‘performance’; Lüdecke *et al*., 2020; Tables S4, S5 and S6 in Supplementary material). Then, GLMMs derived from all potential combinations of included predictors were automatically constructed using the ‘dredge’ function (package MuMIn; Barton, 2009).

All models were compared using the Akaike information criterion corrected for small sample size (AICc), delta AICc (ΔAICc) and the AICc weights (AICcw) (Burnham and Anderson, 2002). A model average was performed, considering all models with delta AICc <2 as relevant (model-average; see Tables S1, S2, S3 in Supplementary Material). The predictors with the highest weights retrieved from the average model were considered as the most relevant, following the indications on Akaike weights by Burnham and Anderson (2002).

## Results

### Patterns in parasite richness

**Fig. 2.**
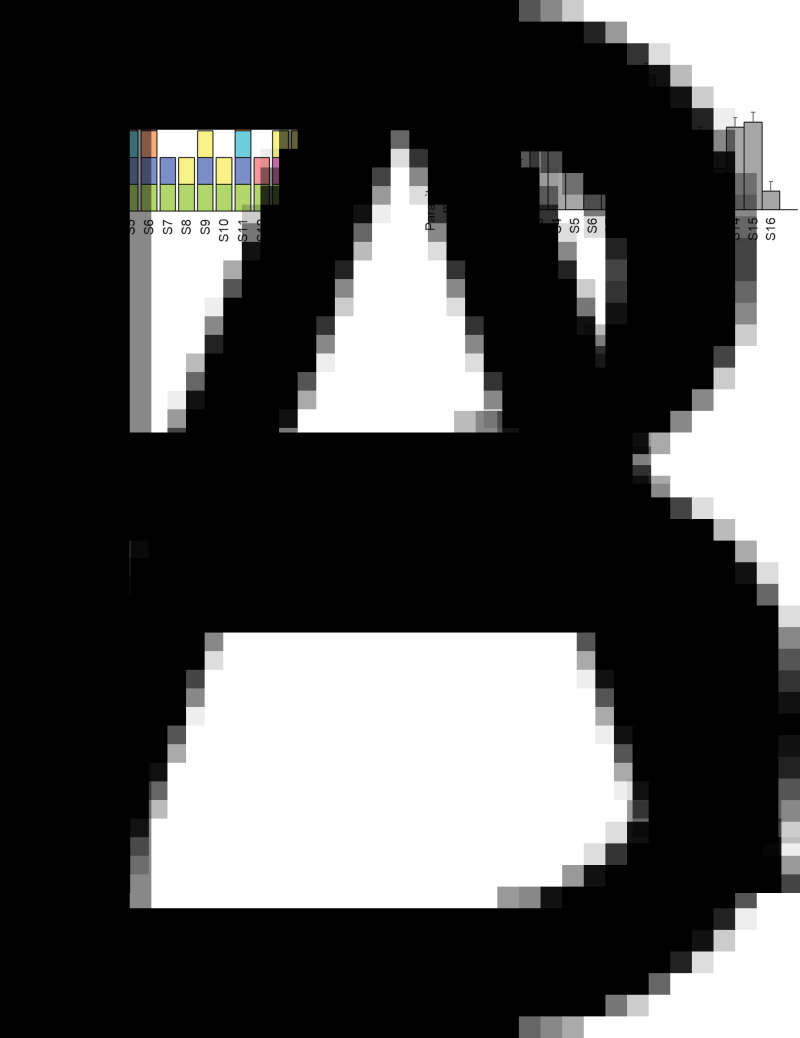
Local parasite richness per station (A) and mean parasite richness per mussel individual (i.e. infra-community richness; B) at each of the 16 sampling stations from east (S1) to west (S16) in the North Adriatic Sea. In (a) the presence of each one parasite species is considered as 1. Error bars in (B) represent the SE.

During the sampling, a total of 320 mussels were collected and dissected. Among sampling locations, total richness ranged from two to seven species or taxa (ciliates, copepods and nematodes), with higher local richness in the western stations (Fig. 2a). Mean species richness per individual mussel at each sampling location ranged from 0.15 to 1.95 (Fig. 2b), and maximum parasite richness per mussel ranged from 0 to 4 species.

### Patterns in parasite prevalence and abundance

**Fig. 3.**
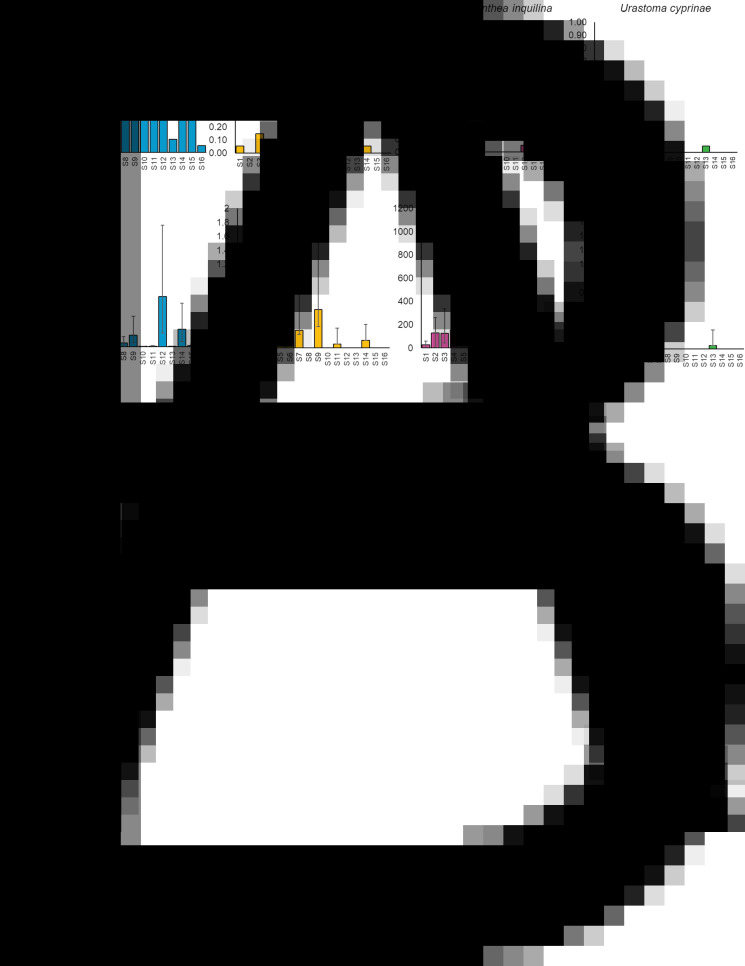
Prevalence (A) and mean abundance (B) of the four most common parasite species found in *Mytilus galloprovincialis* mussels collected during the sampling (*n* = 20 mussels at each station): *Parvatrema duboisi*, *Mytilicola* sp. *Eugymnanthea inquilina* and *Urastoma cyprinae*. Prevalence is calculated as proportion of infected individuals (between 0 and 1). All plots are based on sampling station arranged from east (S1) to west (S16) of the Northern Adriatic Sea. Error bars represent the SE. Notice the different scale for *Mytilicola* sp. and *Urastoma cyprinae* abundance.

A total of seven distinct parasite taxa were detected: metacercariae of the digenean trematode *P. duboisi* [mean prevalence of 0.56; 95% confidence interval (CI): 0.50, 0.61 ± 0.10], the intestinal copepod *Mytilicola* sp. (Mytilicolidae) (0.06; 95% CI: 0.042, 0.097), the hydrozoan *E. inquilina* (0.14; 95% CI: 0.10, 0.18) and the turbellarian *U. cyprinae* (Urastomidae) (0.03; 95% CI: 0.01, 0.05) (Fig. 3a). Other species found were belonging to the nematodes (0.04; 95% CI: 0.02, 0.06), ciliates (0.17; 95% CI: 0.13, 0.22) and copepods (other than *E. inquilina*) (0.03; 95% CI: 0.01, 0.05) groups. However, since genetic data were not available, it cannot be excluded that some of the taxa include cryptic species. Hence, our diversity estimates are probably conservative, especially for ciliates and nematodes. In addition, without genetic data we cannot exclude the possibility that the nematodes we observed are actually free-living species that were only accidently found in the mussels.

*Parvatrema duboisi* displayed the highest abundance, with an average of 68.6 metacercariae 6 (95% CI: 50.74, 93.18) per individual in the total of mussels collected, and a maximum average of 415 metacercariae per individual per station (Fig. 3b, station S12). The highest intensity observed per single infected mussel was approximately 1000 metacercariae.

### Drivers of parasite richness, prevalence and abundance in individual mussels

High collinearity (vif >4) was found between TRIX and salinity in the models of parasite species richness and salinity was selected over TRIX due to its higher contribution to the models. High collinearity was also found in the models of *P. timondavidi* prevalence and abundance, *Mytilicola* sp. prevalence and *U. cyprinae* prevalence, and TRIX was selected over salinity due its higher contribution to the models. In all other models the vif values appeared <4, thus no predictor was excluded (Zuur *et al*., 2010).

Species richness*:* The average model retained when applying the threshold of ΔAICc <2 (see Table S1 in Supplementary Material) included as best explanatory drivers for parasites species richness in individual mussel local richness, host size, host population density and salinity (Fig. S1 in Supplementary Material). The contribution of local richness appeared as the most relevant (Table 2), with the driver positively affecting parasites species richness (Fig. S1 in Supplementary Material).

**Table 2.** Summary table of the relative contribution (Akaike weights) of the drivers to mean parasite richness per mussel individual, prevalence (for *Parvatrema duboisi*, *Eugymnanthea inquilina*, *Mytilicola* sp., *Urastoma cyprinae)* and abundance (for *P. duboisi* and *E. inquilina*), determined based on the average model with a threshold of AICc <2

		Mussel size (mm)	Mussel density (# per 50 × 50 cm plot)	Local parasite richness (*n* of parasite species per station)	TRIX	Salinity
Richness	Species richness per mussel individual	0.76	0.51	**1.00**	–	0.15
Prevalence	*Parvatrema duboisi*	–	0.27	NA	**1.00**	–
	*Eugymnanthea inquilina*	0.17	0.13	NA	**0.85**	0.36
	*Mytilicola* sp.	0.43	0.33	NA	–	–
	*Urastoma cyprinae*	0.28	0.21	NA	**1.00**	–
Abundance	*Parvatrema duboisi*	–	–	NA	**0.63**	–
	*Eugymnanthea inquilina*	0.23	0.16	NA	**0.69**	0.47

*Note*: The drivers included in the model were biotic: mussel size, mussel population density, local richness, and abiotic: salinity and TRIX (indicator of the eutrophication status). The contributions of the most relevant drivers are presented in larger font and in bold. Drivers not added for testing in a model are indicated with NA.

Parasite prevalence: The average model retained for *P. duboisi* prevalence (see Table S2 in Supplementary Material) included TRIX and mussel population density as best explanatory drivers (see Fig. S2a in Supplementary Material). The contribution of TRIX appeared as the most relevant (Table 2), with TRIX positively affecting the prevalence of *P. duboisi* until an optimum of around 4.7 and then turning its effect into negative (Fig. S2a in Supplementary Material). The average model for *E. inquilina* included all drivers and the average model of *U. cyprinae* included all drivers except salinity (Table S2 in Supplementary Material). Yet, in both models the TRIX contribution was the most relevant, with large magnitude of the coefficients (Table 2 and Fig. S2b, d in Supplementary Material). For *E. inquilina* TRIX positively affected prevalence only until an optimum of 3.3, then its effect turned into negative, while for *U. cyprinae* the correlation with prevalence was always negative. For *Mytilicola* sp., the average model included all the drivers (Table S2 in Supplementary Material), however none of the drivers contributed relevantly to the species prevalence (Fig. S2c in Supplementary Material).

Parasite Abundance**:** The average model of *P. duboisi* abundance included only TRIX as driver, while the average model of *E. inquilina* included all the drivers (see Table S3 in Supplementary Material). However, in both average models TRIX appeared as the only relevant driver (Table 2 and Fig. S3 in Supplementary Material). Abundance of *P. duboisi* increased with TRIX until an optimum of 4.4, then decreased.

## Discussion

The present study aimed to assess key drivers of parasite species richness in individual mussels as well as of parasite prevalence and abundance in local mussel populations in the northern Adriatic Sea. Parasite local richness was identified as the main biotic factor driving parasite richness in individual mussels, while eutrophication turned out to be the main abiotic driver for parasite prevalence and abundance. Our results are generally in line with results of previous studies investigating determinants of parasite infection levels in bivalves at large spatial scales (de Montaudouin *et al*., 2009; Studer *et al.*, 2013) and suggest that parasite infection richness and infection levels are the result of a complex interplay of abiotic and biotic factors.

Except for the parasite *P. duboisi*, the species composition found in *M. galloprovincialis* mussels was very similar to the one observed by Rayyan *et al*. (2004) in the Aegean Sea, where the most common species were *E. inquilina*, *U. cyprinae* and *Mytilicola intestinalis*, suggesting a broad distribution range of the different parasite taxa in the Mediterranean. Among these species, *E. inquilina* was recorded in the Eastern Mediterranean Sea only (Piraino *et al*., 1994; Rayyan *et al*., 2004; Mladineo *et al*., 2012) while *U. cyprinae* and *Mytilicola* sp. show a broader distribution, from the Atlantic coast of Portugal to the Spanish coasts until the Black Sea coast (Figueras *et al*., 1991; Claire *et al*., 2010; Özer and Güneydağ, 2014). Their association with mussels ranges from mutualism, with *E. inquilina* ingesting trematodes sporocysts in the host tissue, to parasitism with negative effects on host condition index (Piraino *et al*., 1994; Rayyan *et al*., 2004; Mladineo *et al*., 2012). Other species found in the present study were ciliates, nematodes and other copepods. However, no genetic tools have been employed for confirming the species identified and for assessing the potential presence of cryptic species. Hence, our diversity estimates are probably conservative. Further studies in the Adriatic Sea would need the use of molecular tools to confirm the identity of the different parasite species and to investigate the potential presence of cryptic species.

In our study, the dominant species was *P. duboisi*, with the highest prevalence and abundance in the majority of the sampling locations. This species belongs to the Gymnophallidae family, known to not encyst as other trematode species, and actively ingests its host tissue by oral suckers (Irwin *et al*., 2003; Galaktionov *et al*., 2006). Despite its dominance over our sampling area, this is the first record of *P. duboisi* in the northern Adriatic, and very little information on its spatial distribution and interaction with other species are available (but see Özer and Güneydağ, 2014). The species was identified for the first time in *M. galloprovincialis* in the early 1960s in the Gulf of Marseilles, France, by Bartoli (1965) as *P. timondavidi* (Gymnophallidae), which then became a synonym for *P. duboisi*, the current accepted name (WoRMS, 2021). To our knowledge, no other records followed in the Mediterranean Sea. However, Yanagida *et al*. (2009) found heavily infected individuals of the clam *Ruditapes philippinarum* in the Ariake Sea and lately, a study by Jung *et al*. (2021) confirmed *R. philippinarum* being both, the first and the second intermediate host for *P. duboisi*. During last few decades, this clam spread in the West coasts of the Northern Adriatic Sea, due to the increase of aquaculture activities, and we could argue a spillover mechanism (see Goedknegt *et al*., 2017) might have been generated. Therefore, it would be worth considering further investigations on *R. philippinarum* parasite communities, and comparisons with the one of the native mussel *M. galloprovincialis*.

In our study, parasite species richness in mussel individual was positively correlated with parasite local richness, mussel size, mussel population density and salinity. However, based on the Akaike weights only the contribution of local richness was relevant. The positive correlation between parasite richness in mussel individuals and local richness found in our study is coherent with the review by Poulin (1997), who assessed the infra-community of parasites in the individual host representing a subset of all parasite species occurring in the component community. The infra-community as a subset might be determined by characteristics of the host population, such as the homogeneity among individuals in the susceptibility to infection and the environmental conditions they inhabit (Hartvigsen and Halvorsen, 1994; Poulin, 1997). Mussel size was the second most relevant driver positively contributing to parasite individual richness. The positive correlation with size has been already observed and reviewed in previous studies (Poulin, 2004; Thieltges and Reise, 2006) and is likely the result of a higher chance by different parasites to encounter a larger host, ascribed to its higher movement rate or food uptake (Arneberg, 2002). Furthermore, larger individuals might provide more space and a higher number of niches in their tissue which can by colonized by different parasites (Poulin, 2004).

Interestingly, no correlation between parasite richness in mussel individuals and eutrophication was found, despite the strong correlation among eutrophication and parasite species prevalence and abundance. Previous studies conducted along the German Baltic Sea coast reported eutrophication favouring generalist parasite species or parasites with shorter life cycles (Kesting and Zander, 2000; Zander and Reimer, 2002; Budria, 2017). The higher eutrophication in the central and western coast of the Northern Adriatic Sea due to the presence of many river runoffs may act as a bottleneck for very few species only, limiting the emergence of a proper large-scale pattern for parasite richness. Parasites having *M. galloprovincialis* as intermediate host (i.e. *P. duboisi*) are potentially favoured, since this mussel species is highly adaptable and tolerant to a large variety of environmental conditions (Kovačić et al., 2018). As opposite, the presence of other species may be limited by the absence of hosts or by other combined environmental factors such as the type of substrate, which indirectly influences the density of many infaunal invertebrates (see Poulin and Mouritsen, 2003).

Eutrophication appeared to be the driver contributing most to parasite species prevalence and abundance. *Parvatrema duboisi* prevalence and abundance showed to be significantly higher at intermediate levels of TRIX. The positive effect of eutrophication on the prevalence of *P. duboisi* until medium-eutrophied waters may be explained by an indirect beneficial effect. Hence, previous studies reported that eutrophied and turbid environments might lead to a lower predation risk (Cézilly, 1992; Budria and Candolin, 2014), promoting the survival of infected intermediate hosts and consequently higher transmission rates. This indirect effect might be combined with the benefit of higher nutrient loads, enhancing higher fitness and reproduction of the hosts (Budria and Candolin, 2014; Aalto *et al*., 2015). Nevertheless, our findings also revealed a decrease of *P. duboisi* prevalence when levels of TRIX became higher. Negative effects of high TRIX values were even more substantial for *E. inquilina* and *U. cyprinae* prevalence, as well as *E. inquilina* abundance. Hence, an increase of *E. inquilina* prevalence was detected until TRIX values of 3.3, which still indicate oligotrophic waters. Highly abundant nutrients can indeed induce microalgal blooms producing compounds that may be deleterious for other organisms (Smith and Schindler, 2009) or that lead to hypoxia (Davidson *et al*., 2014), all of which provoke deleterious effects on the host-parasite system (reviewed by Budria, 2017). These negative effects can be even more pronounced in species for which the life cycle is not strictly associated with the host as for trematodes (e.g. *E. inquilina*), which spend a large part of their life as a free-living stage (Kubota, 1983). However, to our knowledge, almost no studies investigated the response of turbellarian prevalence to eutrophication. More information should be gathered about these two overlooked, yet considerably important, species (Kostenko, 2018).

The correlation among TRIX levels and *P. duboisi* abundance in our study was positive until a TRIX of 4.4, which might be explained by the parasite life cycle. Yanagida *et al*. (2009) reported *R. philippinarum* being both the first and second intermediate host of *P. duboisi* in the Ariake Sea, which may be also the case of *M. galloprovincialis*. High nutrient loads available for the single intermediate host can favour a higher parasites production (Johnson *et al*., 2007). Assuming the hosts belonging to the same species and population, a higher parasite production in one mussel host may be followed by a higher chance of infection in a second mussel host inhabiting the very close proximity. This is turn might favour high metacercariae abundance in the second intermediate mussel host.

## Conclusions

In general, among the drivers considered in our study, local parasite richness and TRIX were those mainly contributing to the distribution of parasite richness per mussel individual (with local richness weight of 1.0) and prevalence of the investigated parasites (with TRIX weight of 1.00 for *P. duboisi* and *U. cyprinae* and 0.85 for *E. inquilina*). We detected a pronounced difference between drivers that influenced the tested traits: Biotic drivers (i.e. parasite local richness and mussel size) mainly influenced parasite species richness in mussel individual, while drivers influencing parasite species prevalence and abundance were of mainly abiotic nature (i.e. eutrophication). A very recent study by Friedland *et al*. (2021) reported a decrease in TRIX in the Adriatic Sea over the last few decades, due to an improved nutrient management. Therefore, a decrease in eutrophication could lead to deleterious effects for trematode species such as *P. duboisi*, but may benefit other parasites. However, to better define some TRIX boundaries, a more profound knowledge of the life cycle of the considered species would be needed, as well as *in vitro* studies. The results of our study might be broadened to other similar ecosystems of regions experiencing similar conditions, for example semi-enclosed basins, which due to the presence of many river runoffs and human activities, are subjected to eutrophication phenomena, and in general faster-going effects of global change. During the last few years are the Northern Adriatic Sea provided ‘refugium’ for cold/temperate species (Ben-Rais Lasram *et al*., 2010), including a wide variety of molluscs and marine birds of native and invasive nature. This could lead to new interactions between parasites and their hosts, as well as introduction and propagation of new pathogen species to naïve populations. Further studies should follow and involve other host- and habitat-level drivers as well as molecular techniques for better identifying parasite species. The inclusion in these studies of recently introduced host species and their co-introduced parasites would help our understanding of host-parasite dynamics under global change. Finally, further field survey should take in consideration other important biotic factors for bivalves such as immunity status and should be followed by experimental studies on the effects on important and still overlooked drivers, such as eutrophication.

## Supplementary material

For supplementary material accompanying this paper visit https://doi.org/10.1017/S0031182021001438. click here to view supplementary material
