# Supplementary material for: Biotic and abiotic drivers affect parasite richness, prevalence and abundance in Mytilus galloprovincialis along the Northern Adriatic Sea
Source: Parasitology. 2021 Aug 12;149(1):15–23. doi: 10.1017/S0031182021001438 (PMC8862135; doi:10.1017/S0031182021001438)
Supplement: Supplementary file 1 [file S0031182021001438sup001.docx]

**Supplementary Material**

**Title:** Abiotic and biotic drivers affect parasite richness, prevalence and abundance in *Mytilus galloprovincialis* along the Northern Adriatic Sea

**List of authors:** Claudia Bommarito ^1^, Martin Wahl^1,^ David W Thieltges^2^, Christian Pansch^3^, Matteo Zucchetta ^4^, Fabio Pranovi ^5^

**Affiliations**:

^1^ GEOMAR Helmholtz Centre for Ocean Research Kiel, Department of Marine Ecology; Hohenbergstr. 2, 24105, Kiel, Germany

^2^ NIOZ Royal Netherlands Institute for Sea Research, Department of Coastal Systems, P.O. Box 59, 1790, AB Den Burg Texel, The Netherlands

^3^ Environmental and Marine Biology, Åbo Akademi University, Artillerigatan 6, 20520 Åbo, Finland

^4^ Institute of Polar Sciences, ISP-CNR, Via Torino 155, 30172 Venice-Mestre, Italy

^5^ University Ca’ Foscari of Venice, Department of Environmental Sciences, Informatics and Statistics; Via Torino 155, 30172, Venice, Italy


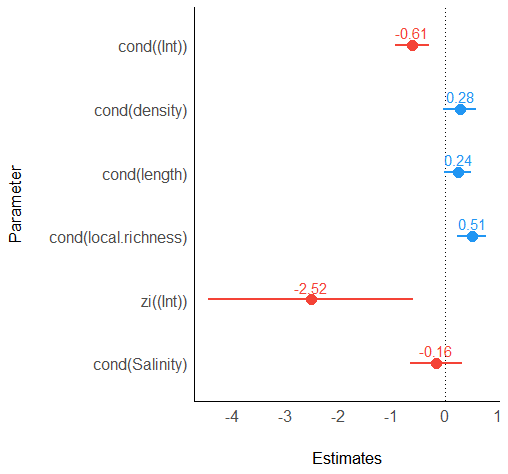


**Figure S1** Estimates of the average models for parasite species richness in mussel individual. The dot whiskers plots show estimated values of the different predictors included in the average model in the y-axis the and the range of the estimates in the x-axis. The whiskers span the 95% of Confidence Interval. The blue whiskers indicate a positive correlation, the red ones a negative correlation between the species prevalence and the predictors.


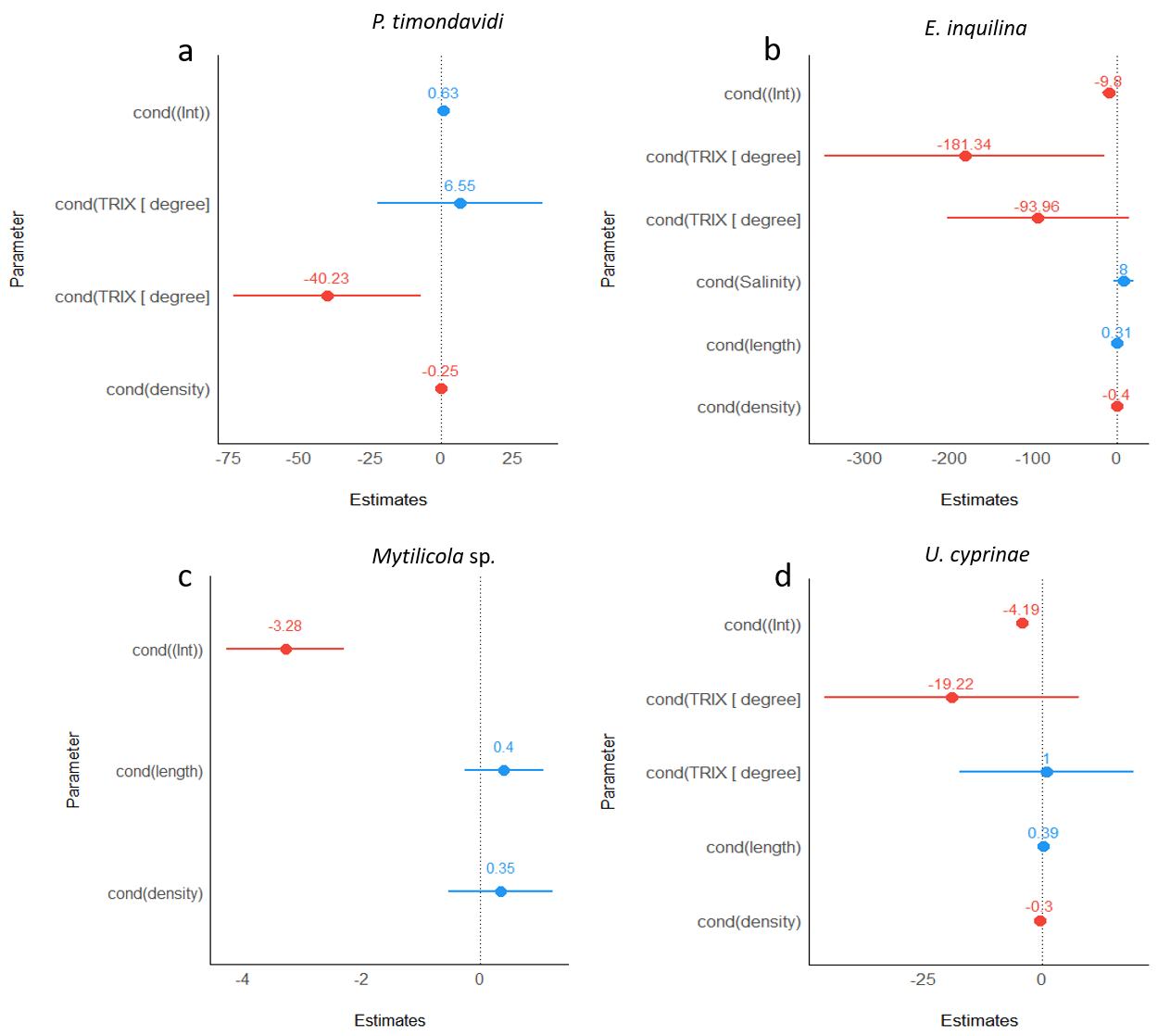


**Figure S2** Estimates of the average models for: a) *Parvatrema duboisi* prevalence, b) *Eugymnanthea inquilina* prevalence, c) *Mytilicola* sp., d) *Urastoma cyprinae*. The dot whiskers plots show the estimates values of the different predictors included in the average model in the y-axis and the range of the estimates in the x-axis. The whiskers span the 95% of Confidence Interval. The blue whiskers indicate a positive correlation, the red ones a negative correlation between the species prevalence and the predictors.

**
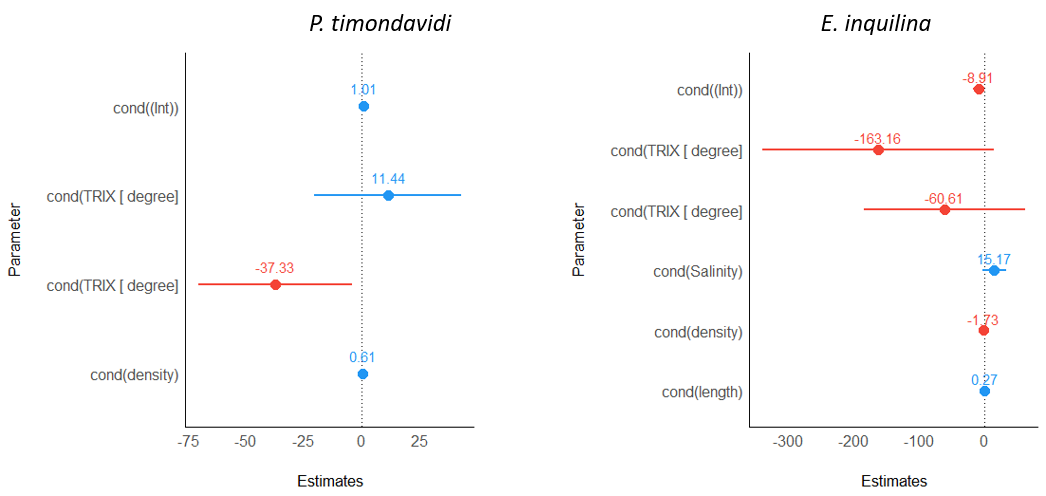
Figure S3** Estimates of the average models for: a) *Parvatrema timondavidi* abundance, b) *Eugymnanthea inquilina* abundance. The dot whiskers plots show in the y-axis the estimates values of the different predictors included in the average model and in the x-axis the range of the estimates. The whiskers span the 95% of Confidence Interval. The blue whiskers indicate a positive correlation, the red ones a negative correlation between the species prevalence and the predictors.

**Table S1** Model selection for the zero truncated Generalized Linear Mixed Model (GLMM) applied in the analysis with parasite richness in individual mussel. Predictors present in the model are indicated with “+” and predictors absent with “-”. Model performance statistics shows the degrees of freedom (df), the transformation applied to the dependent variable and the corrected Akaike Information Criterion (AICc).

| **Dependent Variable** | **Model performance statistics** | | | |  |  |  |  |  |  |
| --- | --- | --- | --- | --- | --- | --- | --- | --- | --- | --- |
|  | TRIX | Salinity | Mussel size | Mussels density | Local richness | df | | delta | AICc | weight |
| Parasite richness in individual mussel | NA | **-** | **+** | **+** | + | 7 | | 0.00 | 617.3 | 0.251 |
|  | NA | - | + | - | + | 6 | | 0.70 | 618.0 | 0.177 |
|  | NA | **-** | **-** | **-** | + | 5 | | 0.78 | 618.1 | 0.170 |
|  | NA | **+** | **+** | + | + | 8 | | 1.69 | 619.0 | 0.108 |
|  | NA | + | + | - | + | 7 | | 2.07 | 619.4 | 0.089 |
|  | NA | - | - | + | + | 6 | | 2.09 | 619.4 | 0.088 |
|  | NA | + | - | - | + | 6 | | 2.57 | 619.9 | 0.069 |
|  | NA | **+** | - | + | + | 7 | | 2.94 | 621.3 | 0.034 |
|  | NA | - | + | - | - | 5 | | 3.03 | 625.3 | 0.005 |
|  | NA | - | - | - | - | 4 | | 3.98 | 626.4 | 0.003 |
|  | NA | + | + | - | - | 6 | | 4.40 | 627.2 | 0.002 |
|  | NA | - | + | + | - | 6 | | 4.75 | 627.3 | 0.002 |
|  | NA | + | - | - | - | 5 | | 5.14 | 627.8 | 0.001 |
|  | NA | - | - | + | - | 5 | | 5.71 | 628.4 | 0.001 |
|  | NA | + | + | + | - | 7 | | 6.40 | 629.2 | 0.001 |
|  | NA | + | - | + | - | 6 | | 7.40 | 629.8 | 0.000 |

**Table S2** Model selection for the Generalized Linear Mixed Models (GLMM, binomial family) applied in the analysis with *Parvatrema duboisi*, *Mytilicola* sp*., Eugymnanthea inquilina and Urastoma cyprinae* prevalence. Predictors present in the model are indicated with “+” and predictors absent with “-”. Model performance statistics shows the degrees of freedom (df), the transformation applied to the dependent variable and the corrected Akaike Information Criterion (AICc).

| **Dependent Variable** | **Model performance statistics** | | | |  |  |  |  |  |
| --- | --- | --- | --- | --- | --- | --- | --- | --- | --- |
|  | TRIX | Salinity | Mussel size | Mussels density | df | | delta | AICc | weight |
| Prevalence *P. timondavidi* | **+** | NA | **-** | **-** | 4 | | 0.00 | 233.9 | 0.395 |
|  | + | NA | - | + | 5 | | 1.99 | 235.9 | 0.146 |
|  | **+** | NA | **+** | **-** | 5 | | 2.03 | 236.0 | 0.143 |
|  | **-** | NA | **-** | **-** | 2 | | 2.20 | 236.1 | 0.131 |
|  | - | NA | - | + | 3 | | 3.67 | 237.6 | 0.063 |
|  | + | NA | + | + | 6 | | 4.04 | 238.0 | 0.052 |
|  | - | NA | + | - | 3 | | 4.24 | 238.2 | 0.047 |
|  | - | NA | + | + | 4 | | 5.71 | 239.6 | 0.023 |
| Prevalence *Mytilicola* sp. | **+** | NA | **-** | **-** | 4 | | 0.00 | 145.3 | 0.213 |
|  | - | NA | - | - | 2 | | 0.19 | 145.5 | 0193 |
|  | + | NA | + | - | 5 | | 0.52 | 145.8 | 0.164 |
|  | - | NA | + | - | 3 | | 0.98 | 146.3 | 0.130 |
|  | + | NA | - | - | 3 | | 1.88 | 147.2 | 0.083 |
|  | + | NA | + | - | 5 | | 2.06 | 147.3 | 0.076 |
|  | + | NA | - | + | 4 | | 2.09 | 147.4 | 0.075 |
|  | + | NA | + | + | 6 | | 2.35 | 147.6 | 0.066 |
| Prevalence *E. inquilina* | **+** | - | **-** | **-** | 4 | | 0.00 | 123.2 | 0.224 |
|  | + | + | - | - | 5 | | 0.91 | 124.1 | 0.142 |
|  | + | - | + | - | 5 | | 1.32 | 124.5 | 0.116 |
|  | - | + | - | - | 3 | | 1.69 | 124.9 | 0.096 |
|  | + | - | - | + | 5 | | 1.92 | 125.1 | 0.086 |
|  | + | + | + | - | 6 | | 2.47 | 125.6 | 0.065 |
|  | + | + | - | + | 6 | | 2.59 | 125.8 | 0.061 |
|  | - | + | + | - | 4 | | 2.99 | 126.2 | 0.050 |
|  | + | - | + | + | 6 | | 3.35 | 126.5 | 0.042 |
|  | - | + | - | + | 4 | | 3.65 | 126.8 | 0.036 |
|  | + | + | + | + | 7 | | 3.99 | 127.2 | 0.030 |
|  | - | + | + | + | 5 | | 4.88 | 128.1 | 0.020 |
|  | - | - | - | - | 2 | | 5.63 | 128.8 | 0.013 |
|  | - | - | + | - | 3 | | 6.38 | 129.6 | 0.009 |
|  | - | - | - | + | 3 | | 7.20 | 130.4 | 0.006 |
|  | - | - | + | + | 4 | | 8.04 | 131.2 | 0.004 |
| Prevalence *U. cyprinae* | **+** | NA | **-** | **-** | **4** | | **0.00** | **77.5** | **0.379** |
|  | + | NA | + | - | 5 | | 1.20 | 78.7 | 0.208 |
|  | **+** | NA | **-** | **+** | 5 | | 1.78 | 79.3 | 0.155 |
|  | - | NA | + | - | 3 | | 2.77 | 80.3 | 0.095 |
|  | + | NA | + | + | 6 | | 3.25 | 80.8 | 0.075 |
|  | - | NA | + | + | 4 | | 4.26 | 81.8 | 0.045 |
|  | - | NA | - | + | 3 | | 5.61 | 83.1 | 0.023 |
|  | - | NA | - | - | 2 | | 5.88 | 83.4 | 0.020 |

**Table S3.** Model selection for the Generalized Linear Mixed Models (GLMM, negative binomial family) applied in the analysis with *Parvatrema duboisi* and*, Eugymnanthea inquilina* abundance. Predictors present in the model are indicated with “+” and predictors absent with “-”. Model performance statistics shows the degrees of freedom (df), the transformation applied to the dependent variable and the corrected Akaike Information Criterion (AICc).

| **Dependent Variable** | **Model performance statistics** | | |  | |  |  |  |  |  |
| --- | --- | --- | --- | --- | --- | --- | --- | --- | --- | --- |
|  | TRIX | Salinity | Mussel size | | Mussel density | | df | delta | AICc |  |
| Abundance *P. timondavidi* | **+** | NA | **-** | | **-** | | 5 | 0.00 | 1950.6 | 0.320 |
|  | **-** | NA | **-** | | **-** | | 3 | 1.06 | 1951.7 | 0.189 |
|  | + | NA | - | | + | | 6 | 2.05 | 1952.7 | 0.115 |
|  | - | NA | - | | + | | 4 | 2.07 | 1952.7 | 0.114 |
|  | + | NA | + | | - | | 6 | 2.08 | 1952.7 | 0.113 |
|  | - | NA | + | | - | | 4 | 3.11 | 1953.8 | 0.068 |
|  | - | NA | + | | + | | 5 | 4.13 | 1954.8 | 0.041 |
|  | + | NA | + | | + | | 7 | 4.14 | 1954.8 | 0.040 |
| Abundance *E. inquilina* | **+** | **-** | **-** | | **-** | | 5 | 0.00 | 756.7 | 0.169 |
|  | - | + | - | | - | | 4 | 0.42 | 757.1 | 0.136 |
|  | + | - | - | | + | | 6 | 0.81 | 757.5 | 0.113 |
|  | + | + | - | | - | | 6 | 0.90 | 757.6 | 0.107 |
|  | + | - | + | | - | | 6 | 1.38 | 758.1 | 0.084 |
|  | - | + | + | | - | | 5 | 1.62 | 758.3 | 0.075 |
|  | - | + | - | | + | | 5 | 2.21 | 758.9 | 0.056 |
|  | + | + | + | | - | | 7 | 2.27 | 759.0 | 0.054 |
|  | + | - | + | | + | | 7 | 2.35 | 759.1 | 0.052 |
|  | + | + | - | | + | | 7 | 2.69 | 759.4 | 0.044 |
|  | - | + | + | | + | | 6 | 3.53 | 760.2 | 0.029 |
|  | - | - | - | | - | | 3 | 3.62 | 760.3 | 0.028 |
|  | + | + | + | | + | | 8 | 4.18 | 760.9 | 0.021 |
|  | - | - | + | | - | | 4 | 4.94 | 761.7 | 0.014 |
|  | - | - | - | | + | | 4 | 5.30 | 762.0 | 0.012 |
|  | - | - | + | | + | | 5 | 6.66 | 763.4 | 0.006 |

**Table S4** VIF values of the zero truncated Generalized Linear Mixed Model (GLMM) applied in the analysis with parasite species richness in mussel individual. The NA indicates the factors not included in the model.

| Variable | TRIX | Salinity | Mussel density | Mussel length | Local richness |
| --- | --- | --- | --- | --- | --- |
| Parasite species richness in mussel individual | NA | 1.64 | 1.44 | 1.24 | 1.62 |

**Table S5** VIF values of the Generalized Linear Mixed Models (GLMMs) following binomial distributions applied for *Parvatrema duboisi, Mytilicola* sp*., Eugymnanthea inquilina and Urastoma cyprinae* prevalence and including all the fixed factors (TRIX, salinity, mussel density and mussel size (length)). The NA indicates the factors not included in the model.

| Species | TRIX | Salinity | Mussel density | Mussel length |
| --- | --- | --- | --- | --- |
| *P. timondavidi* | 1.28 | NA | 1.04 | 1.28 |
| *Mytilicola* sp. | 1.11 | NA | 1.24 | 1.16 |
| *E. inquilina* | 1.31 | 2.63 | 2.36 | 1.10 |
| *U. cyprinae* | 1.67 | NA | 1.77 | 1.64 |

**Table S6** VIF values of the Generalised Linear Mixed Models (GLMMs) following negative binomial distributions applied for *Parvatrema timondavidi* and *Eugymnanthea inquilina* abundance and including all the fixed factors (TRIX, Salinity, mussel density and mussel size (length)). The NA indicates the factors not included in the model.

| Species | TRIX | Salinity | Mussel density | Mussel length |
| --- | --- | --- | --- | --- |
| *P. timondavidi* | 1.19 | NA | 1.19 | 1.01 |
| *E. inquilina* | 1.38 | 2.53 | 2.18 | 1.03 |
